# Vasculitis and vasculopathy associated with inborn errors of immunity: an overview

**DOI:** 10.3389/fped.2023.1258301

**Published:** 2024-01-31

**Authors:** Silvia Federici, Bianca Laura Cinicola, Francesco La Torre, Riccardo Castagnoli, Vassilios Lougaris, Giuliana Giardino, Stefano Volpi, Roberta Caorsi, Lucia Leonardi, Stefania Corrente, Annarosa Soresina, Caterina Cancrini, Antonella Insalaco, Marco Gattorno, Fabrizio De Benedetti, Gian Luigi Marseglia, Michele Miraglia Del Giudice, Fabio Cardinale

**Affiliations:** ^1^ Division of Rheumatology, Bambino Gesù Children’s Hospital, IRCCS, Rome, Italy; ^2^ Department of Maternal Infantile and Urological Sciences, Sapienza University of Rome, Rome, Italy; ^3^ Department of Molecular Medicine, Sapienza University of Rome, Rome, Italy; ^4^ Department of Pediatrics, Giovanni XXIII Pediatric Hospital, University of Bari, Bari, Italy; ^5^ Pediatric Unit, Department of Clinical, Surgical, Diagnostic, and Pediatric Sciences, University of Pavia, Pavia, Italy; ^6^ Pediatric Clinic, Fondazione IRCCS Policlinico San Matteo, Pavia, Italy; ^7^ Department of Clinical and Experimental Sciences, Pediatrics Clinic and Institute for Molecular Medicine A. Nocivelli, University of Brescia and ASST-Spedali Civili di Brescia, Brescia, Italy; ^8^ Pediatric Section, Department of Translational Medical Sciences, Federico II University, Naples, Italy; ^9^ Center for Autoinflammatory Diseases and Immunodeficiency, IRCCS Istituto Giannina Gaslini, Genoa, Italy; ^10^ Division of Pediatrics, S. Camillo-Forlanini Hospital, Rome, Italy; ^11^ Unit of Pediatric Immunology, Pediatrics Clinic, University of Brescia, ASST-Spedali Civili Brescia, Brescia, Italy; ^12^ Department of Systems Medicine, University of Rome Tor Vergata, Rome, Italy; ^13^ Academic Department of Pediatrics, Immune and Infectious Diseases Division, Research Unit of Primary Immunodeficiencies, Bambino Gesù Children’s Hospital, IRCCS, Rome, Italy; ^14^ Department of Woman, Child and of General and Specialized Surgery, University of Campania ‘Luigi Vanvitelli’, Naples, Italy

**Keywords:** autoinflammatory diseases, DADA2, SAVI, monogenic lupus, haploinsufficiency A20, vasculopathy

## Abstract

Systemic autoinflammatory diseases (SAIDs) are disorders of innate immunity, which are characterized by unprovoked recurrent flares of systemic inflammation often characterized by fever associated with clinical manifestations mainly involving the musculoskeletal, mucocutaneous, gastrointestinal, and nervous systems. Several conditions also present with varied, sometimes prominent, involvement of the vascular system, with features of vasculitis characterized by variable target vessel involvement and organ damage. Here, we report a systematic review of vasculitis and vasculopathy associated with inborn errors of immunity.

## 1 Introduction

The term “autoinflammatory” was coined in 1999 to differentiate this group of disorders from autoimmune syndromes, which are mainly driven by adaptive immunity and are characterized by autoreactive T cells and autoantibodies. Systemic autoinflammatory diseases (SAIDs) initially included only the periodic fever syndromes, but the number of conditions in this group is growing rapidly. Vasculitis may be a prominent feature accompanying SAIDs. In this review, we focus on monogenic autoinflammatory diseases in which vascular involvement represents a key feature (Table 1).

**Table 1:** Vascular involvement in monogenic autoinflammatory diseases.

	Inheritance	Gene	Chromosome	Mutated protein	Vascular involvement
SAVI	AD	*TMEM173*	5q31.2	STING	Small vessels vasculitis
DADA2	AR	*ADA2*	22q11	ADA2	Medium and small- vessels vasculitis mimicking the pattern seen in polyarteritis nodosa with highly variable clinical expression
HA20	AD	*TNFAIP3*	6q23.3	A20	Variable vessel vasculitis resembling Behçets disease

## 2 STING-associated vasculopathy with onset in infancy

STING-associated vasculopathy with onset in infancy (SAVI) is a rare autoinflammatory disease first reported in 2014 (1). It is characterized by an early onset of systemic inflammation associated with a small vessel vasculopathy, leading to severe skin lesions and pulmonary and joint involvement.

*De novo* or autosomal dominant gain-of-function mutations in the transmembrane protein 173 (*TMEM173*) gene, encoding STimulator of INterferon Genes (STING), lead to constitutive STING activation and upregulation of type 1 IFN production. A total of 19 mutation sites have been reported in the literature to date, mainly substitution, with p.Val155Met being the most prevalent (https://infevers.umai-montpellier.fr/web/).

STING is expressed by several cell types among which are endothelial cells, skin cells, hematopoietic cells, bronchial epithelial cells, and alveolar cells. Consequently, multiple tissues are affected.

### 2.1 Pathogenesis

The innate immune system plays a key role in protecting the host against microbiological agents. Pattern recognition receptor (PRR) proteins, expressed mainly by cells of the innate immune system, can recognize pathogen-associated molecular patterns (PAMPs) associated with microbial pathogens and damage-associated molecular patterns (DAMPs) associated with host cell elements that are released during cell damage or death.

The recognition of a ligand by a PRR triggers a signaling cascade that, in turn, leads to the expression of immune genes. Most PRRs can discriminate between self and non-self in a highly conserved and tightly regulated mechanism that defends the host against microbiological pathogens while avoiding attacks on the self. *TMEM173* encodes STING, a key signaling molecule in cytosolic DNA-sensing pathways. Upon activation in response to cytosolic DNA, STING dimerizes and induces the phosphorylation of transcription factors IRF3 and NF-κB, leading to their translocation to the nucleus and the production of IFNs and inflammatory cytokines. Secreted type I IFN acts in an autocrine and paracrine way by binding the interferon-α receptor (IFNAR), which in turn activates several downstream signaling pathways, most notably the Janus kinase (JAK)-signal transducer and activator of transcription (STAT) pathway.

Structural modeling in SAVI demonstrates the constitutive activation of the mutated STING protein due to stabilized dimerization. This was confirmed by Jeremiah et al., who found that STING was mostly localized, at a steady state, in the Golgi and perinuclear punctiform vesicles of the patient's fibroblasts, which was previously reported to correspond with STING activation (2). Upon 2′3′-cGAMP stimulation, the mutant STING remained localized in the same structures confirming its activation in patient cells independently from ligand addition.

### 2.2 Clinical manifestations

Disease onset is usually in the first weeks of life, rarely after 1 year of age. Initial symptoms often include intermittent low-grade fever, recurrent cough, and failure to thrive. Moreover, patients usually present with progressive interstitial lung disease (ILD) and cutaneous chilblain lesions.

In a recent systematic review of the literature, respiratory symptoms (tachypnea, dyspnea, cough, and milk choking) or skin manifestations associated with growth failure were the presenting features in 35% and 57% of patients, respectively (3).

The most typical skin lesions are erythematous–purpuric patches and plaques on cold-sensitive areas mainly the cheeks, nasal tip, ears, and acral sites. Cutaneous manifestations typically worsen after exposure to cold and may progress to painful ulcerations with eschar formation and tissue loss. Histological characterization shows a dermal inflammatory infiltrate with features of leukocytoclastic vasculitis and microthrombotic angiopathy of small dermal vessels (1).

Lung involvement should be sought in case of respiratory symptoms, even if it may be found in asymptomatic patients. Nail clubbing may be an early sign of ILD and may appear before other pulmonary symptoms.

On CT scan, ILD may present as ground glass areas, cysts, reticulations, interlobular septal thickening, or pleuritis. Additional features such as consolidations, bronchiectasis, emphysema, lymphadenopathy, and pulmonary hypertension may be found. Liu et al. (1) described a scattered mixed lymphocytic inflammatory infiltrate, interstitial fibrosis, and emphysematous changes in the lung biopsies of two patients. Pulmonary function tests demonstrate a severe restrictive pattern with a decreased diffusing capacity for carbon monoxide (DLCO).

The clinical spectrum of disease manifestations has expanded since the first description, and patients may present with polyarthritis, myositis, kidney, brain, thyroid involvement, photosensitivity, and hair loss, demonstrating important phenotypic variability. Moreover, three patients with exclusive pulmonary disease (4, 5) and 16 patients with only cutaneous involvement (1, 6–8) have been reported.

This variability seems to be partially related to the different genotypes, and patients carrying variants outside the dimerization domain of the protein appear to have more atypical phenotypes (9).

Jeremiah et al. (6) speculated that intrafamilial phenotypic differences may be related to modifying genes acting downstream of STING or to viral infections that promote IFN signaling.

During disease flares, SAVI patients usually display increased acute phase reactants (C-reactive protein and erythrocyte sedimentation rate). Moreover, some patients may have transiently positive or low-titer autoantibodies [antinuclear antibodies (ANA), RF], and the majority of patients present with hyperimmunoglobulinemia, mainly IgG.

T-cell and natural killer (NK) lymphopenia are consistent features of the disease; however, patients usually do not present with recurrent infections.

### 2.3 Treatment

The therapeutic approach of SAVI patients is often challenging, and the prognosis is poor, especially in the case of severe lung disease with a high mortality early in life. Glucocorticoids are only partially effective and are burdened with important side effects for prolonged therapies due to the development of steroid dependence. Moreover, there has been minimal success with the use of other disease-modifying therapies, such as methotrexate, mycophenolate mofetil, antimalarials, infliximab, and rituximab (1, 10, 11).

According to the evidence of a prominent pathogenetic role of constitutive type I IFN signaling, attempts with JAK inhibitor drugs to block the JAK-STAT pathway at the IFNAR receptor have been made.

The first report of a suppressive effect of JAK inhibition on type I IFN signaling came from Liu et al. (1) who demonstrated a sustained blockade of constitutive STAT1 phosphorylation following the treatment of blood mononuclear cells (PBMC) in a patient with SAVI with tofacitinib, ruxolitinib, or baricitinib.

Subsequently, other case reports showed a good response to JAK inhibitors in children with SAVI. Frémond et al. (12) described three children aged 5–12 years, two of whom had prominent pulmonary involvement and systemic inflammation, and one a prevalent skin involvement, treated with ruxolitinib, a JAK1/2 inhibitor. All patients experienced a pronounced improvement in general condition, a reduction of fever episodes, an almost complete resolution of skin manifestation, and an amelioration of pulmonary function. All of them were able to taper off the glucocorticoids.

The same encouraging results with ruxolitinib were described by Volpi et al. (13) in three children aged 3–13 years, with cutaneous and pulmonary involvement associated with recurrent fever and growth failure and renal involvement in one child. Ruxolitinib was associated with a general improvement in skin and pulmonary disease and was able to resolve the microhematuria. All patients were able to taper glucocorticoids. However, one patient displayed multiple severe viral infections after starting therapy, suggesting that these drugs may significantly increase this risk.

Tofacitinib significantly improved skin lesions in a 9-year-old child with SAVI, but did not ameliorate pulmonary involvement after two months of treatment. This was attributed to already-established lung damage (14). In another case report, Balci et al. (15) reported a 6-month-old child who failed previous treatment with ruxolitinib responding to baricitinib, another JAK1/2 inhibitor.

A larger cohort of 18 patients affected with SAVI (4 patients), CANDLE (10 patients), and a CANDLE-like disease (4 patients) were treated with baricitinib for a mean duration of 2.3 years under a compassionate use protocol (16). The patients experienced a significant amelioration of general conditions, demonstrated by a reduction in mean autoinflammatory diary scores as well as glucocorticoid doses by at least 50% from baseline. The most common adverse events in these patients were upper respiratory tract infections and BK viruria. The clinical significance of low positive BK titers was not clear, and serum and urine BK titers along with renal function were regularly monitored. Among the SAVI patients, three-fourths presented with respiratory tract infections, all presented with mucocutaneous infections, and two-fourths had osteomyelitis.

Altogether, these findings demonstrate that JAK inhibition may be an effective therapeutic strategy for SAVI, although the clinical response may be different and some features (i.e., pulmonary involvement) only partially respond to the treatment. This may be due to already-established organ damage prior to the initiation of the therapy or to the involvement of other pathways contributing to the damage. In this regard, Luksch et al. (17) demonstrated that a SAVI-associated STING mutation can determine a pulmonary disease independently of type I IFN in mice.

In conclusion, although promising, future clinical trials are necessary to fully understand the therapeutic effect and the safety profile of long-term JAK inhibition with particular regard to the risk of infections or malignancies.

## 3 Deficiency of adenosine deaminase 2

Deficiency of adenosine deaminase 2 (DADA2) is a complex monogenic autoinflammatory disease first described in 2014 by two separate groups as a syndrome of recurrent fever, livedo racemosa, early-onset strokes, and peripheral vasculopathy resembling polyarteritis nodosa (PAN) (18, 19).

It is caused by biallelic loss-of-function mutations in the *ADA2* gene. Over 100 variants, mainly missense, have been reported in all structural domains of the protein (Infevers).

### 3.1 Pathogenesis

Adenosine deaminase (ADA) is a ubiquitously expressed metabolic enzyme involved in purine metabolism. Humans express two enzymes, namely, ADA1 and ADA2, that catalyze the deamination of adenosine and 2'-deoxyadenosine to inosine and deoxyinosine, respectively. ADA2 has been previously considered as an isozyme of ADA1, but they are different in structure, substrate affinity, cellular localization, and expression.

Cellular damage stimulates adenosine formation. Its effect on cellular activity and local inflammation depends on several factors such as local concentration, receptor expression, receptor type, and affinity. Upon binding to four different cell surface receptors (A1, A2A, A2B, and A3), either a decrease or increase in intracellular cyclic AMP is triggered that, in turn, influences cellular activation through multiple biological pathways.

ADA1 is the major ADA expressed in almost all cells in humans. It reduces the amount of adenosine in the intracellular space. Mutant ADA1 causes a severe combined immunodeficiency (SCID) resulting from the effect of toxic metabolites accumulated in developing lymphocytes, with consequent increased B- and T-cell apoptosis.

Conversely, ADA2 is an extracellular homodimer. It is secreted by activated monocytes, macrophages, and dendritic cells. In a physiologic state, ADA2 is present at low concentrations with low affinity for adenosine. It is not known whether it has a role in controlling the extracellular homeostasis of adenosine under these conditions. However, in stress conditions, ADA2 seems to play an important role in the degradation of extracellular adenosine at the site of inflammation produced by excessive ATP breakdown.

Apart from the deaminase activity, ADA2 seems to have a growth factor activity on the development of endothelial and hematopoietic cells (19–21).

The formation of NETs by neutrophils (NETosis) is an important instrument of the innate immune system to fight infections, and it has been demonstrated to cause inflammation in different autoimmune conditions such as systemic lupus erythematosus (SLE) and antineutrophilic cytoplasmic antibody (ANCA)-associated vasculitis (22, 23).

Carmona-Rivera et al. recently demonstrated *in vitro* that an increased amount of adenosine in the extracellular space in the absence of ADA2 can stimulate NET formation.

DADA2 patients have higher levels of plasma adenosine and an increased number of circulating low-density granulocytes that are primed to undergo NETosis.

NETosis also stimulates activated macrophages to produce large amounts of TNF-α. This observation is important because it provides a novel mechanistic link between a defective ADA2 function and inflammatory cytokine production, in addition to a rationale for the use of TNF inhibitors in DADA2 patients with an inflammatory/vasculitis phenotype. It is still not clear whether NETosis and NET-induced TNF-α also occur in DADA2 patients presenting with a predominantly hematological or immunodeficient clinical phenotype.

However, it has been speculated that increased TNF-α levels may be responsible for the hematological manifestations of the disease, as TNF-α may have a role in bone marrow failure (BMF) in patients with aplastic anemia (24).

### 3.2 Clinical manifestations

At the time of the description, inflammatory vasculitis was the hallmark of the disease. Nowadays, the spectrum of clinical manifestations has significantly broadened, and DADA2 patients presenting with immunodeficiency or hematologic manifestations are increasingly described. Sometimes these features may even predominate the clinical phenotype.

As in other autoinflammatory diseases, patients may complain of constitutional symptoms, such as recurrent fever, weight loss, failure to thrive, fatigue/malaise, myalgia, and arthralgia.

Both inter- and intrafamilial phenotypic variability can be observed, which mainly depends on the residual enzymatic activity. Nowadays this can be measured and may be of great help, especially in those patients with atypical clinical phenotypes or non-confirmatory genetic analyses.

In general, patients with the vasculitis phenotype usually carry variants that determine a residual ADA2 activity up to 40%–60% of the normal range (hypomorphic mutations) unlike patients with a prominent hematological phenotype in which the residual enzymatic activity is usually <3% (25).

However, some individuals carrying two pathogenic ADA2 mutations and with absent residual enzymatic activity may remain asymptomatic until late in life or never present clinical manifestations of the disease (26). On the other hand, it may happen that some carriers of pathogenic ADA2 mutations with enzymatic activity levels in the carrier range develop mild and/or late-onset features of the disease. Moreover, heterozygous individuals may present symptoms of the disease, among which are vascular strokes (19, 27) underscoring the need for additional studies to determine the clinical impact of ADA2 haploinsufficiency.

In addition to the amount of residual enzymatic activity, a possible genotype/phenotype correlation has been suspected following the identification of different disease patterns. Recently, Lee (25) analyzed a cohort of 167 DADA2 patients classified into three groups based on the main phenotype: 100 with a vasculitis/stroke phenotype, 38 with pure red cell aplasia (PRCA), and 29 with BMF. In total, 61 different mutations were identified, 45 of which were fairly uniquely associated with one group (21 to the vasculitis group, 12 to the PRCA group, and 12 to the BMF group). Only two mutations were described as associated with all three different phenotypes, thus suggesting that some variants are mostly associated with a specific phenotype.

In agreement with the first published papers, a typical vascular involvement with features of vasculitis or vasculopathy is described in more than 75% of patients involving the skin brain, gastrointestinal tract, and renal vessels.

The involvement of medium and small vessels is similar to that seen in panarteritis nodosa. Skin biopsies reveal medium-vessel vasculitis or leukocytoclastic vasculitis that may determine amputation when involving arteries supplying the extremities (18, 28). Not surprisingly, many DADA2 patients were previously diagnosed as having PAN. Caorsi et al. (28) performed the genetic analysis for *ADA2* in 48 children diagnosed with PAN in Italy. Fifteen patients turned out to carry biallelic *ADA2* mutations. When compared clinically, DADA2 patients displayed an earlier disease onset and more frequent skin and neurologic manifestations.

Pathogenic *ADA2* mutations have been found in patients with Sneddon syndrome and one child diagnosed with HHV-8-negative Castleman disease (29).

Skin is involved in nearly 90% of cases. Livedo racemosa is the most typical manifestation found in up to 73% of patients. It is usually subtle and patchy and mainly localized to the lower limb, even if it may present in the upper limb or the torso (26). Subcutaneous nodules/edema, erythema-multiforme–like lesions, ulcers, and less commonly non-specific erythematous maculopapular rash and urticarial and psoriasiform rash have also been described (30–32).

Neurologic involvement of DADA2 is found in 50%–77% of patients (26, 33–36).

Ischemic strokes are usually localized in the brainstem, thalamus, basal ganglia, and internal capsule (37). They may represent the initial features of the disease and may be recurrent (38, 39). The majority are small lacunar infarcts but larger ones, causing permanent neurologic impairments and sometimes death, may develop (36). Over time, the recurrence of these small, sometimes undetectable strokes, can determine a severe neurologic impairment (40).

The occurrence of transient ischemic attacks without radiological findings (41) and ischemic lesions on MRI in patients with normal cerebral angiography suggest a prevalent small-vessel involvement of the CNS (39, 42, 43).

Vascular spasms in the absence of inflammation may also contribute to the development of strokes (42).

Consequently, ADA2 deficiency should be considered despite normal inflammatory markers and/or evidence of cerebral vasculitis and *ADA2* mutation screening should be conducted in children with otherwise unexplained ischemic stroke.

**Figure 1:**
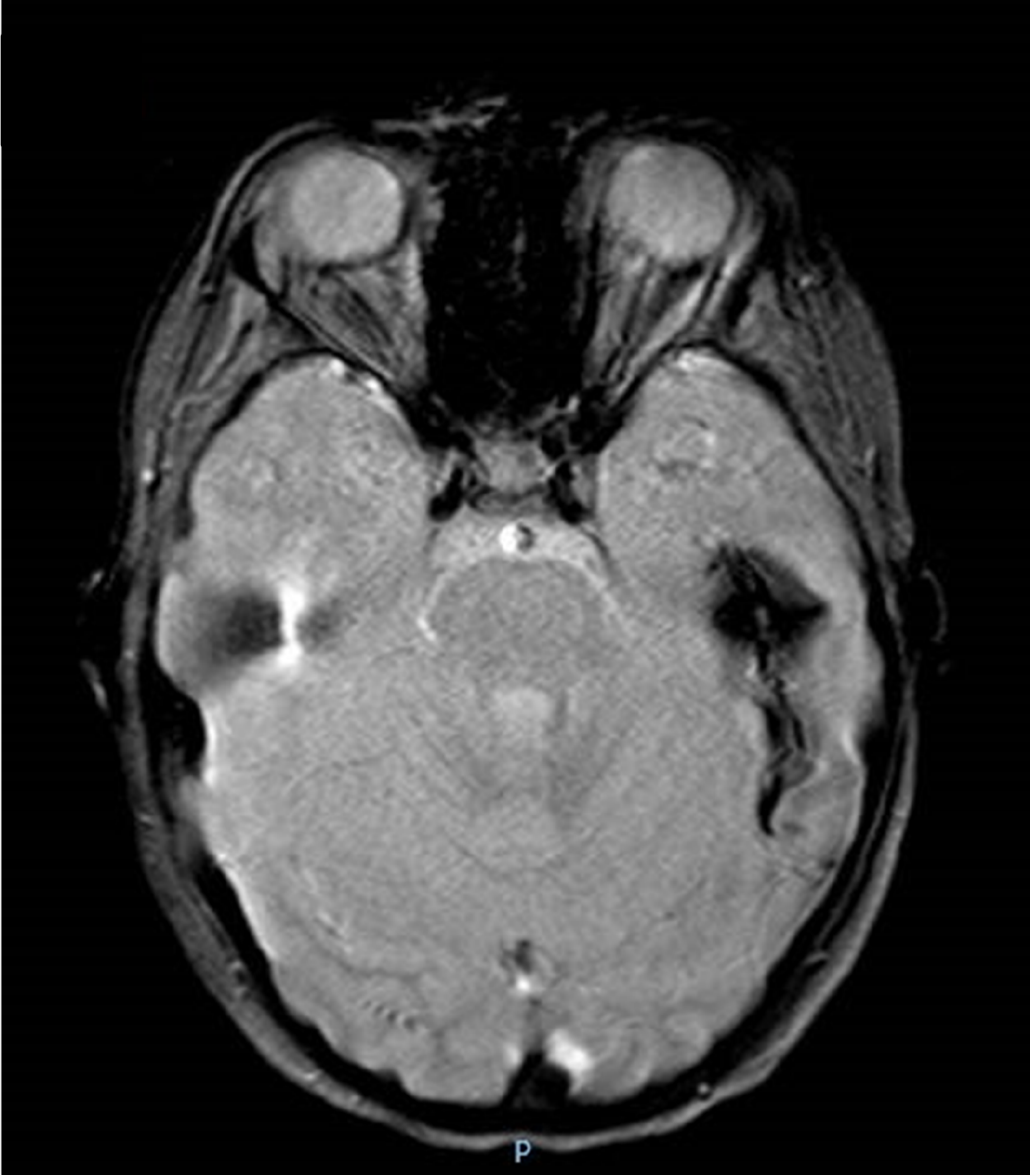
Colliquated poromalacic area in the deep left temporal region, at the site of the previous hemorrhage, surrounded by a thick hemosiderin rim.

Hemorrhagic strokes have also been reported (Figure 1). Zhou et al. (19) revealed in the cerebral biopsy of two patients with intracranial hemorrhage and the absence of signs of cerebral vasculitis, the presence of extravasal red blood cells from small vessels.

A wide range of neuropathies may be found in DADA2 patients (28). Cranial nerve (CN) palsies and neurosensory hearing loss have been reported. In some cohorts, peripheral neuropathy is described in up to 50% of cases, and spastic paraplegia has been reported as a presenting symptom of the disease (28, 44). Ophthalmologic involvement including vision loss, central retinal artery occlusion, optic nerve atrophy, uveitis, diplopia, nystagmus, and strabismus has also been reported (19, 36).

Bone marrow dysfunction, which can include pure red blood cell aplasia (PRCA) with transfusion dependence resembling Diamond–Blackfan anemia (34), isolated refractory thrombocytopenia, and leukopenia, is now recognized as a prominent and sometimes isolated manifestation of the disease (34, 45).

Leukopenia is found in approximately 60% of cases and involves both the myeloid and lymphoid lineage. Neutropenia, reported in 10%–50% of cases (46), may be quite severe leading to recurrent infections and abscess formation. Functional studies do not reveal any neutrophil impairment; conversely, a recent paper describes their increased propensity to netosis and activation in patients with the autoinflammatory phenotype (47). Lymphopenia involving both the B and T compartments is present in approximately 10% of patients with B cells usually being more affected. Finally, the NIH group described a case of severe monocytopenia associated with mild neutropenia in a patient who was thought to have GATA2 haploinsufficiency (48). These hematologic defects are often combined with multiple cases of pancytopenia.

A mild immunodeficiency with low IgM levels has been described since the initial description of the disease (19). In a paper from Schepp et al. (49), 11/181 screened patients with antibody deficiencies and unknown genetic defects with or without vascular manifestations turned out to carry two *ADA2* mutations with decreased ADA2 plasma activity. In more than 50% of these patients, recurrent infections, rather than the classic features of inflammatory vasculitis, were the presenting manifestation of the disease.

A low immunoglobulin (Ig) level is caused by both B-cell intrinsic and extrinsic factors. In a recent paper, Schena et al. (50) confirmed that DADA2 patients have a reduced total number of CD27 positive memory B cells compared to healthy subjects and that, within the memory compartment, the number of class-switched memory B cells is also decreased. Defective class-switching may be partly due to the functional impairment of T follicular helper (TFH) cells, but it cannot be responsible by itself for deficient IgM production. A reduction in the number of antibody-producing cells is probably a contributing factor. Schepp et al. demonstrated an inverse correlation between C-reactive protein and Ig levels in a patient, suggesting that inflammation may directly act on the B-cell compartment (49).

Apart from TFH impairment, no other T-cell function defects are reported. Infection may then be caused by a quantitative reduction due to generalized lymphopenia.

Both viral and bacterial infections are frequent in DADA2 patients with antibody deficiencies (49), whereas fungal and mycobacterial infections are rare and limited to cases with severe reduction in the number of white blood cells (34, 48).

The concurrence of DADA2 and lymphoproliferative diseases has been also reported (40, 46, 51, 52).

As vasculitis is a main feature of the disease, other organs with dense vascularization may be affected, among which are the gastrointestinal and renal systems.

Among the gastrointestinal symptoms, abdominal pain and chronic gastritis are often reported. Vasculitis may lead to intestinal necrosis, bowel perforation, and arterial stenosis (33, 53). Liver biopsies may show evidence of nodular regenerative hyperplasia and/or hepatic sclerosis, potentially leading to end-stage liver disease (40).

Renal aneurysm and infarction have been reported in some patients (18, 26, 36). Hypertension can be seen independent of other renal involvement and may contribute to the development of stroke and intracranial hemorrhage (26, 28, 33).

Finally, some other inflammatory manifestations have been described, including pleuritis, pericarditis, myocarditis, meningitis, and amyloidosis (18, 19).

An increased type I IFN score (IS) has been demonstrated in some patients with DADA2. In 2018, Insalaco et al. analyzed the IS in 5 patients with DADA2 and 10 healthy donors (54). The authors found some degree of correlation between disease severity and the elevation of the IS and a reduction/normalization of the IS score after the introduction of TNF inhibitors therapy, concluding that the IS score may be used as a biomarker of disease activity, severity, and response to treatment in DADA2.

Recently, multidisciplinary consensus statements for the evaluation and management of DADA2 patients have been developed (55).

### 3.3 Treatment

Early treatment is very important to prevent potentially devastating complications. During flares, high-dose systemic glucocorticoids are effective in most cases, but the majority of patients have refractory or relapsing disease upon tapering (28).

TNF inhibition has been demonstrated to be highly effective in patients with a predominant vasculitic/inflammatory phenotype, resulting in a control of inflammation, reduction/resolution of skin rash and hepatosplenomegaly, the occurrence of a catch-up growth, and an improvement of red blood cell and platelet counts due to relief of the inflammatory burden.

However, the most important effect of this therapy seems to be the dramatic protection against strokes. After a few case reports, this was confirmed in a study by the NIH group, in which a retrospective analysis of a cohort of 15 DADA2 patients showed a total of 37 strokes, all occurred prior to the initiation of TNF inhibitors vs. none post-treatment with a median follow-up of 47 months (range, 25–128). Among TNF inhibitors, etanercept, adalimumab, or infliximab are the most used (18, 19, 26, 33), but there is not enough data to support the use of one over the other. In those cases not achieving a complete clinical response, an increase of the dosage or a switch from a TNF inhibitors to another is possible.

TNF inhibitors may be associated with intravenous immunoglobulin replacement and antimicrobial/antiviral drugs in those patients with low serum immunoglobulins and recurrent infections (49).

Given the genetic nature of the disease TNF inhibitors should be continued for life because of the risk of relapse after therapy discontinuation, especially in those with vasculitis and CNS manifestations (56). Conversely, TNF inhibitors seem not to be as effective in rescuing severe bone marrow abnormalities.

Hematopoietic stem cell transplantation (HSCT) can be curative, returning plasma ADA2 activity to normal levels in individuals with BMF and/or immune dysregulation that is non-responsive to TNF inhibition.

After initial case reports (41, 48, 57), this was well demonstrated in one study in which 14 patients with bone marrow dysfunction or immunodeficiency (6 of whom also had vasculitis and 3 intracranial hemorrhages obtained a complete resolution of the hematologic and immunologic phenotype after HSCT. In about half of the cases, a DADA2 diagnosis was made after transplant, the indication of which was respectively PRCA, neutropenia, immune-mediated thrombocytopenia, and pancytopenia (45).

Finally, initial studies focusing on understanding the potential role of gene therapy in DADA2 patients are ongoing.

Regarding asymptomatic individuals with biallelic *ADA2* pathogenic variants, there are no predictors that future strokes or other disease manifestations might occur. Because of the potentially devastating consequences of strokes, patients who have biallelic *ADA2* pathogenic variants should probably be treated with TNF inhibitors.

## 4 Monogenic Behçet and haploinsufficiency A20 (HA20)

Behçet's disease (BD) is a rare condition of unknown etiology, with an estimated prevalence of 1–2/100,000 inhabitants in Europe (58). It is an inflammatory disease with a chronic course and a multisystemic involvement classified among vasculitis. In BD, both arterial and venous vessels of all sizes may be affected. The most common clinical manifestations are oral and genital ulcers, erythema nodosum, arthralgia, and uveitis. In some cases, BD can be life-threatening, especially when large vessels are involved (59–61). In children, the diagnosis is based on the pediatric criteria published in 2016's “Pediatric Behçet Disease” (PEDBD) (62). The disease is more frequent in populations from the ancient Silk Road territories connecting the Far East to the Mediterranean Sea and is strongly associated with the HLA B51 antigen. Several works published almost 30 years ago already speculated on a possible mendelian transmission in some families with early-onset BD (63–65). Recently, mutations in the *TNFAIP3* gene have been associated with HA20 and monogenic vasculitis with an early onset resembling Behçet disease. The first description of the disease was published by Zhou et al. (66) in 2016.

### 4.1 Pathogenesis

HA20 is caused by loss-of-function mutations in *TNFAIP3* coding A20 protein. These result in a reduced suppression of NF-κB activity, and as an enhancement of nucleotide-binding domain-like receptor protein (NLRP3) inflammasome activation. Both of these pathways determine an overproduction of proinflammatory cytokines, among which IL-1β, IL-6, IL-18, and TNF-α (67–69).

A total of 75 variants have been described in the *TNFAIP3* gene, mostly classified as pathogenic or likely pathogenic (Infevers). Disease-causing variants are inherited in an autosomal dominant pattern or may be *de novo*. A20 protein, also known as tumor necrosis factor alpha-induced protein 3 (TNFAIP3), is encoded by the *TNFAIP3* gene. It is composed of an N-terminal ovarian tumor domain (OTU) followed by 7 zinc finger domains (ZnFs) including 790 amino acid residues (70). The OTU domain has a deubiquitinating activity for k63-linked ubiquitin chains, whereas the zinc finger (ZnF) recognizes k63-linked ubiquitin chains. This region is also crucial for A20 E3 ligase activity and dimerization to add k48-linked ubiquitin chains, and it accelerates protein degradation in the proteasome (71, 72).

A20 both removes ubiquitin chains bound to K63 thanks to its OTU domain (deubiquitination) and attaches ubiquitin chains to K48 (ubiquitination). The former destabilizes protein substrates, and the latter accelerates their degradation in proteasome. Through these activities, A20 suppresses NF-κB activity by targeting substrates such as NF-κB essential modifier (NEMO), receptor-interacting serine/threonine kinase 1 (RIP1), and TNF receptor-associated factor 6 (TRAF6) for proteasomal degradation (73, 74). In mice, A20 deficiency leads to insufficient downregulation of NF-κB activity and dysregulation of the NLRP3 inflammasome, both NF-κB dependent and NF-κB independent (75–78). Recently, Rajamäki reported a novel caspase-8-dependent mechanism linking reduced A20 function to enhanced NLRP3 inflammasome activation in the HA20 patients’ immune cells (79).

The broad expression of A20 in different cells of the immune system (dendritic cells, B cells, T cells, and macrophages) means that this protein plays a key role in immune dysregulation (80). B-lineage deletion of A20 perturbs lymphoid homeostasis and leads to an autoimmune disease resembling SLE (81).

### 4.2 Clinical manifestations

Patients affected by HA20 present with recurrent fevers, oral, and/or genital ulcers, gastrointestinal manifestations, skin rash, polyarthritis, and neurological symptoms. HA20 is frequently misdiagnosed as BD. However, it differs from classical BD in terms of early age at onset, a less common eye involvement, a higher incidence of recurrent fever, a poor association with HLAB51, and an overrepresentation of gastrointestinal symptoms (82–85). Additionally, unlike typical BD, some papers recently reported HA20 patients presenting with a predominantly autoimmune picture including thyroiditis, diabetes, rheumatoid arthritis, SLE, and autoimmune lymphoproliferative syndrome (ALPS) (68, 86–88).

The severity of the disease varies greatly regardless of the genotype (67). In a recent systematic review of the literature about published cases of HA20, the authors collected 61 cases (62% female) from 26 families carrying a heterozygous loss-of-function mutation in *TNFAIP3*. The clinical presentation was very different, even between members of the same family carrying the same mutation. Disease onset was at a mean age of 14 years with two-thirds of patients having their first symptoms before the age of 10. All patients initially received another diagnosis, including Behcet's disease, juvenile idiopathic arthritis or rheumatoid arthritis, PFAPA syndrome, autoimmune thyroiditis, Crohn's disease, SLE, and adult-onset Still disease (68). Oral and/or genital ulcers were reported in two-thirds of the patients. Almost half of them presented recurrent episodes of fever, resembling other inflammatory conditions, a skin rash (ranging from non-specific rash, erythema nodosum-like lesions, psoriasis, folliculitis, pustules, to malar rash), and/or gastrointestinal symptoms, including abdominal pain, vomiting, diarrhea, abdominal lymphedema, and intestinal edema. One-third of patients had musculoskeletal manifestations, such as arthralgia or arthritis while only a few patients displayed an ocular involvement.

As in inflammatory bowel diseases (IBD) or BD, the gastrointestinal involvement may be extensive with ulcers diffusing along the entire gastrointestinal tract and signs of inflammation on endoscopy. In some cases, patients may present digestive life-threatening hemorrhages possibly leading to death (82–84).

The finding of autoantibodies in some patients may be non-specific and unrelated to an autoimmune disease or, conversely, associated with defined autoimmune diseases such as SLE, Hashimoto thyroiditis, or type 1 diabetes.

HA20 patients may also present other clinical symptoms, among which are cardiovascular manifestations, nephrotic syndrome, vasculitis, and respiratory tract infections.

### 4.3 Treatment

The use of glucocorticoids, colchicine, TNF inhibitors, and IL1 blockade has been reported. Response to colchicine, a first-line therapy in classical BD, is inconstant and unpredictable, and pharmacological control of inflammation may not be easy (69). Colchicine has been reported as not or inadequately effective in more than half of the patients who have required glucocorticoids or other immunosuppressive agents (methotrexate, ciclosporin, thalidomide) (82). A biological treatment as second-line therapy has frequently been used mostly TNF inhibitors, IL1 inhibitors, IL 6 inhibitors, and B-cell-depleting therapies (anti-CD20) (82). In non-responsive patients, JAK inhibitors or HSCT have been used (69, 82).

## 5 Monogenic lupus

SLE is a complex disease whose etiology is not entirely understood. Genetic and epigenetic factors and immunological defects seem to be involved in its development (89). Studies on twins demonstrated a strong genetic risk of SLE (90, 91).

Recently, genome-wide association studies have helped to identify rare inherited pathogenic variants in a single gene with high penetrance associated with SLE and lupus-like phenotypes, leading to the definition of monogenic lupus (92).

### 5.1 Clinical manifestations

This form refers to a specific subset of SLE patients characterized by distinct genetic abnormalities, early-onset of the disease (usually at <5 years of age), and a broad range of clinical symptoms (93). Among them, mucocutaneous alterations, particularly chilblain lesions and central nervous system (CNS) diseases, are prominent and may overlap with other diseases such as monogenic interferonopathies (94). Juvenile-onset systemic lupus erythematosus (JSLE) and monogenic lupus can manifest with vasculitis. JSLE-related vasculitis symptoms may be non-specific and affect different organs, including the CNS (95).

### 5.2 Mechanisms and specific presentations

Upregulated IFN-I signaling represents a hallmark of SLE, and the persistent exposure of nuclear material to nucleic acid sensors is one of the critical risk factors (96). Indeed, the detection of apoptotic debris and nucleic acids activates nucleic acid sensing pathways, such as those involving Toll-like receptors (TLR) or cytosolic sensors, leading to excessive production of interferon *α* and the development of autoimmunity (97).

Monogenic lupus is associated with the alteration of three main pathways: (1) defects in the clearance of apoptotic bodies and self-derived nucleic acids such as complement deficiencies and mutations in extracellular DNase genes; (2) IFN signaling pathway defects involving either nucleic acid-sensing or the production and response to IFNs; and (3) B-cell and T-cell tolerance defects (98) (Table 2).

**Table 2:** Monogenic lupus.

	Pathway	Gene	Inheritance	Clinical manifestation
Defects in the clearance of apoptotic bodies and self-derived nucleic acid	*Complement deficiency*	C1q	AR	SLE with nephritis, neurological involvement, photosensitivity, skin rash, recurrent infections, AGS
		C4	AR	SLE with multiorgan involvement, nephritis, skin rash, recurrent infections, AGS
		C1r/C1s	AR	SLE with skin rash, nephritis, recurrent infections, AGS
		C2	AR	Mild SLE with photosensitivity, arthritis; mild or absent renal, neurologic, or pleuropericardial involvement; AGS
		C3	AR	SLE with malar rash, photosensitivity, arthralgia/arthritis, Raynaud's phenomenon, recurrent infections
	*Defects in nucleic acid degradation*	Dnase I	AD	SLE, Sjogren syndrome
		Dnase 1L3	AR	SLE with nephritis, hypocomplementemic urticarial vasculitis syndrome
		Dnase II	AR	SLE, autoimmune manifestations (severe cytopenias, hepatosplenomegaly, cholestatic hepatitis, nephritis)
Defects in IFN signaling pathways: SLE-like type I interferonopathies	*Defects in nuclease activity*	TREX1 (Dnase III)	AD	Familiar chilblain lupus, AGS, SLE with severe cerebrovascular disease
		SAMHD1	AR	Familiar chilblain lupus, AGS, SLE with cerebrovascular disease
		RNASEH2A, RNASEH2B, RNASEH2C	AR	AGS, FCL, SLE
		ADAR1	AR/AD	AGS, FCL, SLE
	*Defects in a negative regulator of IFN signaling*	ISG15	AR	AGS, SLE, MSMD
		USP18	AR	AGS, SLE, MSMD
	*Constitutive activation or enhanced sensitivity of innate immune sensors*	IFIH1/MDA5	AD	AGS, SLE, FCL, Singleton Merten Syndrome
		RIG1	AR	AGS, SLE
		TMEM173/STING	AD	STING-associated vasculopathy, FCL, SLE
B-cell and T-cell tolerance	*Defects in B-cell stimulation and proliferation*	PRKCD	AR	SLE with skin and renal involvement, lymphoproliferation, cerebral vasculitis
	*Defects in TCR genes rearrangement and recombination*	RAG1	AR	SLE/SCID
		RAG2	AR/AD	
	*Defects in apoptosis*	FAS/FASL	AD	SLE/ALPS

SLE, systemic lupus erythematosus; AGS, Aicardi–Goutières syndrome; NPSLE, neuropsychiatric SLE; FCL, familiar chilblain lupus; MSMD, Mendelian susceptibility to mycobacterial disease; TCR, T-cell receptor; SCID, severe combined immunodeficiency; ALPS, autoimmune lymphoproliferative disease; ADAR1, RNA-specific ADA 1.

#### 5.2.1 Clearance of apoptotic bodies and self-derived nucleic acids defects

##### 5.2.1.1 Complement deficiency

Among these rare diseases, the first cause of monogenic SLE is related to genetic defects in the complement system.

Complement is a crucial part of the innate immune system, acting as a lytic agent against pathogens and as an opsonin to remove apoptotic bodies and autologous antigens or immune complexes, thus reducing or preventing vascular deposition.

Moreover, complement can decrease type I interferon production by plasmacytoid dendritic cells and have a role in the regulation of adaptive immunity (99).

Any defect in one of the complement components may cause a loss of tolerance, the accumulation of autoantigens, lymphoid cell activation, and ultimately systemic inflammation (100).

Inherited defects in early complement proteins (C1q, C1r/C1s, C2, C3, and C4) are related to a high risk of developing SLE. The risk is 90% in patients with C1q deficiency, 65% in C1r/C1s deficiency, 75% in C4 deficiency, and 10% in C2 deficiency (101).

Patients affected by complement deficiencies generally develop early-onset SLE (102). The main features of the disease are severe cutaneous manifestations and a high mortality rate due to recurrent infections.

Approximately 93% of C1q deficiency patients develop lupus-like manifestations, including photosensitive skin rash, nephritis, oral ulceration, arthritis, and cerebral disease. Moreover, these patients present normal levels of C3 and C4, high ANA, and negative anti-dsDNA (103, 104).

Deficiencies of C1r and C1s are rare and associated with a high mortality rate for recurrent and severe infections. Very few lupus-like disease cases have skin involvement and ANA positivity (105).

Homozygous C2 deficiency is the most frequent hereditary deficiency among the classical pathway complement alterations, with a prevalence of 1:10,000–20,000 in the population. However, only 10%–30% of patients develop SLE (106).

The main features of this kind of defect are early-onset infections, arthritis, photosensitive skin involvement, positive ANA, negative anti-dsDNA, and positive extractable nuclear antigen antibodies (ENA) (mainly anti-Ro/SSA) (107).

Homozygous C3 deficiency is a rare disease, and SLE is not a common manifestation. It is characterized by recurrent severe infections because of the role of a cleavage product of C3 (C3b) in the opsonization of bacteria (100). Signs of lupus, such as fever, rash, glomerulonephritis, and arthritis, are described in up to 28% of the patients (108).

C4 deficiency is strongly associated with the lupus phenotype (106). C4 is encoded by two different genes, C4A and C4B, located on chromosome 6. There exist 2–8 copies of the C4A and C4B genes with an intricate gene copy number variation pattern. A higher copy number of C4 genes is a protective factor, while lower gene copy numbers of total C4 and C4A are considered risk factors associated with SLE (109). The main characteristics of these SLE patients are early disease onset, skin involvement, lupus nephritis ANA, and ENA (mainly anti-Ro/SSA) positivity (107, 110).

##### 5.2.1.2 Defects in nucleic acid degradation

Deoxyribonucleases (DNase) are enzymes that catalyze the degradation of DNA molecules. Recessively inherited loss-of-function mutations in one of the DNase genes lead to insufficient clearance and accumulation of endosomal, cytosolic, or extracellular DNA (111) that stimulates the activation of intracellular nucleic acid sensors, such as TLR7 and 9, promoting excessive type I IFN production, inflammation, and the occurrence of SLE-like manifestations (112).

To date, four different DNases, DNase I, DNase1L3, DNase II, and DNase III, also called TREX1, have been linked to monogenic and early-onset lupus. The first three are characterized by ANA and anti-dsDNA antibody positivity and low levels of complement in the serum (113).

DNase I is a critical serum endonuclease for degrading extracellular dsDNA from dying cells.

This mutation represents an infrequent cause of lupus (114). Four SLE cases have been reported so far with a mutation in DNAse I (115, 116) that displayed very high levels of anti-dsDNA.

DNase1L3 encodes for one of the three homologs of DNase I enzymes, playing an important role in the clearance of DNA debris from apoptotic cells and exogenous DNA and of neutrophil extracellular traps (NETs) (117).

Mutations of this nuclease cause a fully penetrant autosomal recessive form of SLE characterized by defective DNA degradation. DNase1L3 alterations were identified in patients with familial SLE (118), characterized by early disease onset, a variable degree of renal involvement, positive ANA and anti-dsDNA, ANCA, and low complement fractions C3 and C4. Hypocomplementemic urticarial vasculitis syndrome, a disease characterized by recurrent urticarial, cutaneous vasculitis, arthritis, and glomerulonephritis, was described in a family with three children affected by the LOF mutation in DNase1L3 (119).

DNase II is a major lysosomal endonuclease necessary to cleave exogenous DNA from apoptotic cells within macrophage phagosomes (117).

LOF DNaseII alterations were reported in three children with neonatal onset of disease and severe autoimmune manifestations such as severe cytopenia, hepatosplenomegaly, cholestatic hepatitis, and proteinuria resembling a membranous glomerulonephritis. One patient developed deforming arthritis (120).

#### 5.2.2 Defects in IFN signaling pathways: SLE-like type I interferonopathies

This term refers to Mendalian disorders characterized by exacerbated type I IFN expression.

The upregulation of the type 1 IFN pathway may happen through three different mechanisms: defects in nuclease activity [TREX1, SAMHD1, RNases, RNA-specific ADA 1 (ADAR1)], alteration of a negative regulator of IFN signaling (ISG15, USP18), and constitutive activation or enhanced sensitivity of innate immune sensors (MDA5, RIG-I, STING).

Vasculopathy of the skin with chilblain lesions and systemic vasculitis are common clinical features across type I interferonopathies. Aicardi–Goutières syndrome (AGS) and familial chilblain lupus (FCL) are the prototypes of these monogenic diseases (95).

DNase III, also called TREX1, plays an important role in the metabolism of cytosolic single- and double-stranded DNA (121). Autosomal dominant or recessive inheritance patterns exist and are related to variable disease expression, based on the extent of the effect of the disease-causing variant on DNase degradation activity (122).

Patients with SLE carry mutations in TREX1 in up to 2% of cases (123, 124).

Alteration of the same gene has been linked to the two related disorders, FCL and AGS.

The first is a rare form of chronic cutaneous lupus, with an autosomal dominant inheritance and early onset, and is characterized by cold-induced vasculitic skin lesions of the extremities, high titers of multiple autoantibodies, and hypergammaglobulinemia (125). Prevalence data is not available, but a few TREX1-associated FCL have been described (126).

TREX1 mutations, mostly biallelic, are also detected in 24% of AGS patients (127). This condition is characterized by infantile-onset neurologic manifestations, including encephalopathy with basal ganglia calcifications and white matter lesions, cerebrospinal lymphocytosis mimicking congenital viral infections, and progressive neurologic involvement (128) associated with chilblain-like lesions (127, 129, 130). Associations between AGS and early-onset SLE have been reported (131–134). AGS subjects carrying TREX1 mutations can present with SLE manifestations such as cytopenia, cutaneous lesions, oral ulcers, and arthritis, as well as positive autoantibodies (ANA, ENA, and anti-dsDNA) (135).

Mutations in other genes encoding a cytosolic nucleic acid sensor and nucleic acid processing enzymes, such as SAMHD1, RNase H2, and ADAR1, are also responsible for AGS (136).

Recessively inherited LOF mutations in the SAMHD1 gene prevent the degradation of the DNA precursors, resulting in increased levels of deoxyribonucleoside triphosphates (dNTPs), impaired DNA replication and impaired repair mechanisms, ultimately leading to DNA damage, cell cycle arrest, and death (137). As for TREX1 mutations, pathogenic variants in the SAMHD1 gene have been associated with SLE, AGS, and FCL (135, 138).

RNase H2 is ubiquitously expressed; degrades RNA:DNA heteroduplexes, and plays a role in ribonucleotide excision repair. Biallelic LOF mutations in RNASEH2A, RNASEH2B, RNASEH2C, and ADAR1 result in the accumulation of RNA molecules and consequently an overactivation of type I IFN signaling (139). Alterations of these genes cause a spectrum of SLE and AGS phenotypes with a wide range of severity and overlapping features (136, 140, 141).

#### 5.2.3 Defective B-cell and T-cell tolerance

Self-tolerance is a highly regulated process, maintained at both central and peripheral levels. Examples of self-tolerance mechanisms are apoptosis, B-cell receptor editing, and suppression by regulatory T cells (142). Failure of self-tolerance can result in the development of autoimmunity, as in the case of SLE (143).

Protein kinase C-delta (PRKCD) and recombination-activating 1 or 2 genes (RAG1/2) are crucial factors in establishing self-tolerance.

The PRKCD gene encodes protein kinase C-δ (PKC-δ), which is important in the control of cell proliferation and apoptosis. This protein promotes a negative selection of B cells and regulates T-cell proliferation. PKC-δ deficiency results in autoreactive B-cell development and excessive T-cell activation, contributing to the development of T-cell autoimmunity (144).

Few cases of homozygous mutation in PRKCD have been reported. They all demonstrate early-onset disease and typical lupus features, including autoantibody production, nephritis, and skin involvement (145–149). Moreover, lymphoproliferation and, in some cases, susceptibility to infections can be observed, generally described as impaired apoptosis and expansion of immature B cells.

Disruption of B-cell tolerance and the development of lupus manifestations have also been reported in patients with recombination-activating 1 or 2 genes (RAG1/2) mutations.

RAG1/2 is an essential enzyme for the V(D)J recombination and the generation of BCR to TCR variability (150). LOF RAG mutations lead to SCID. At the same time, hypomorphic mutations (pathogenic variants in heterozygous states) of the same gene are associated with leaky/atypical SCID, characterized by forms of immune dysregulation including systemic autoimmunity (151).

A classical SLE patient with a heterozygous RAG2 mutation has been described as presenting with erosive polyarthritis, serositis, skin involvement, Raynaud phenomenon, Sicca syndrome, and lupus nephritis. The patient also had a history of recurrent infections, high titers of ANA and dsDNA, Smith, RNP, histone, SSA, cardiolipin, leukopenia, and hypocomplementemia (152). Compound heterozygous RAG1 mutations have also been identified in a family with the combined immunodeficiency phenotype, autoimmune cytopenia, and multiple autoantibodies, including anti-IFNα antibodies (153).

Finally, alteration of the regulatory mechanisms of apoptosis and nuclear debris clearance contributes to increased autoantigen exposure and autoantibody production, resulting in the development of autoimmunity and SLE manifestations.

The FAS cell surface death receptor (FAS) is a protein belonging to the TNF receptor superfamily. The binding with its ligand FASL activates complex signaling aimed at the regulation of apoptosis (154, 155). Autosomal dominant mutations in the FAS–FASL apoptotic pathway fail to remove autoreactive and dead cells and result in a lymphoproliferative syndrome with autoimmune features (ALPS), including SLE (156, 157).

Polymorphisms in the FAS and FASL genes seem to increase susceptibility to SLE (158, 159), and ALPS patients may satisfy the criteria for SLE (160). The presence of autoimmune cytopenia and autoantibodies are the most common features but, in some patients, skin involvement, renal disease, arthritis, and serositis are also described (156, 161).

### 5.3 Treatment

NGS and WES elucidated the genetic causes of SLE and helped identify monogenic lupus as a specific category of disease. These advances in the understanding of SLE pathogenesis may promote, in the future, novel target therapeutic options and personalized treatments to cure the disease. At present, despite these advancements, the management of monogenic lupus is still a challenge.

Regarding nuclear debris clearance defects, non-specific conventional therapeutic agents, including glucocorticoids and immunosuppressive drugs, are commonly used (162). There are case reports of effective therapy with fresh frozen plasma (FFP) to replace complement components in the cases of C2 and *C1q*-deficient SLE patients (163–165). However, it is challenging to consider a regular FFP infusion as a standard therapeutic option. In other small case series, HSCT has been reported as a promising treatment in *C1q*-deficient SLE patients (166, 167), but risks related to this treatment should always be considered.

B-cell targeting therapies may represent attractive options in B-cell–related monogenic lupus.

Belimumab is a monoclonal humanized IgG antibody that inhibits BAFF, a B-lymphocyte stimulator causing a reduction in the number of B cells and regulating their function. Favorable results were observed in adults with active SLE, particularly those with cutaneous disease and arthritis. Recently, belimumab has been approved for patients with cSLE older than 5 years of age, but its use in monogenic SLE is uncommon. However, it was recently administered to six patients with refractory monogenic SLE (five *C1q* deficiency and one DNase II deficiency) combined with conventional treatment. The main indications for belimumab administration were refractory mucocutaneous manifestations, arthritis, renal manifestations, and failure to decrease glucocorticoids. The belimumab infusion was well-tolerated, with variable clinical responses (168).

Rituximab (146) and ofatumumab (169) were effective in siblings with PKC-δ deficiency.

Moreover, considering the fundamental role of elevated type I interferon expression in lupus pathogenesis, new therapies targeting the IFN pathway in SLE patients could also be beneficial for some monogenic forms.

Hydroxychloroquine is one of the most commonly administered drugs because of its ability to downregulate the type I IFN signature (170), and it has been demonstrated useful in a patient with PKC-δ deficiency (149). Recently, anti-IFNa and anti-IFNAR monoclonal antibodies have been proposed and are currently under study for the treatment of SLE patients (143, 171, 172).

Last, several JAK inhibitors are under clinical trials in SLE patients, and they also may be promising for monogenic SLE (173, 174).

#### 5.3.1 Immune dysregulation

The association between primary immune deficiencies (PID) and vasculitis has been extensively described.

Among PIDs, immune diseases secondary to actin cytoskeletal dysfunction cause inflammatory manifestations, including vasculopathies. The Arp2/3 complex is one of the major protein complexes with a role in actin polymerization and cellular motility. Defects in the Arp2/3 genes coding for this complex's regulatory subunits cause different syndromes.

The Wiskott–Aldrich syndrome protein (WASp) is a member of the actin nucleation-promoting factor (NPF) family, which is involved in the transduction of signals from cell surface receptors to the actin cytoskeleton through the actin-related protein (Arp)2/3 complex. WASp is encoded by the WAS gene and is a key regulator of the cytoskeletal organization of hematopoietic cells via actin polymerization (175).

Wiskott–Aldrich syndrome (WAS) is a rare X-linked primary immunodeficiency caused by mutations in the WAS gene, characterized by the triad of microthrombocytopenia, eczema, and recurrent infections. It is a progressive combined immunodeficiency that predisposes to an increased risk of autoimmunity and malignancies (176). In particular, autoimmunity is reported in approximately 40% of the patients, and the most common manifestations are hemolytic anemia, autoimmune neutropenia, vasculitis, arthritis, IgA nephropathy, and inflammatory bowel disease. Some patients can develop multiple autoimmune manifestations. Schönlein-Henoch diseases like purpura, dermatomyositis, recurrent angioedema, and uveitis have also been reported in some patients (177).

Vasculitis is a known complication of WAS and may account for life-threatening bleeding. It can be relatively frequent in patients before 2 years of age (178). Milder forms of vasculitis are frequent in WAS, but this condition can also be systemic or affect different organs such as the lungs, brain, bladder, kidneys, and gut (178–181).

A vascular involvement of the aorta or its branches has previously been reported in WAS. A 24-year-old man was described as having an aneurysmal dilatation of branches of the hepatic and superior mesenteric arteries and the kidneys, caused by necrotizing vasculitis (182). A 23-year-old patient with idiopathic chronic aortitis, characterized by lymphocytic and neutrophilic infiltrates, developed ascending and descending thoracic aneurysms requiring surgical correction (183). A 21-year-old male developed a destructive, full-thickness, chronic aortitis that led to aortic root dilatation, which was treated with an aortic valve and root replacement (184).

Treatment with glucocorticoids, in association with cyclosporine, is usually effective in treating vasculitis (177).

Among the regulatory subunits of the Arp2/3 complex, actin-related protein 2/3 complex subunit 1 (ARPC1) is a crucial molecule driving the movement of the cytoskeleton and thus essential for actin filament branching. In humans, it exists in two isoforms, namely, ARPC1A and ARPC1B, with the second prominently expressed in blood cells (185).

Platelet abnormalities with eosinophilia and immune-mediated inflammatory disease (PLTEID) are associated with biallelic loss-of-function mutations of the ARPC1B gene (186). It is clinically similar to WAS in that it is characterized by systemic inflammation with lymphoproliferation and immunodeficiency. The main laboratory features are impaired T-cell migration and proliferation, increased levels of immunoglobulin E (IgE) and IgA, eosinophilia, and thrombocytopenia. To date, only a few individuals have been identified with the ARPC1B deficiency syndrome worldwide (187). The disease presents with a very early clinical onset, usually with severe infections, eczema, food allergies (anaphylactic reactions), and asthma. The main autoimmune features are cutaneous leukocytoclastic vasculitis and inflammatory bowel disease (188–191) HSCT is considered a curative treatment option for these patients (188).

TAP deficiency is a monogenic disorder affecting MHC class I–restricted surface expression, and it resembles granulomatous polyangiitis.

The transporter associated with antigen processing (TAP) protein complex is a member of the ATP-binding cassette transporter family. It translocates cytosolic molecules into the endoplasmic reticulum (ER), where they bind major histocompatibility (MHC) class I molecules, which are in turn recognized by T cells or NK cells after exposition at the cell surface (192).

TAP deficiency usually manifests with recurrent upper and lower respiratory tract bacterial infections and granulomatous skin ulcers. These cutaneous lesions occur in approximately half of the patients and mainly appear in the second decade (193). These lesions present with a polyangiitis-like phenotype and may mimic Wegener's granulomatosis (194). Other manifestations include leukocytoclastic vasculitis and retinal vasculitis (195, 196). The pathophysiology is not entirely understood. It is hypothesized that previous infections may induce the accumulation of NK and γδ T cells in the cutaneous tissue (195, 196). Before starting the treatment, a differential diagnosis should be made carefully because, in these patients, immunosuppressive therapy is useless and, in some cases, harmful because it can be associated with the progression of lesions (197).

Vasculitis is described in SAP deficiency, where CNS vasculitis with aneurysmal dilatation is the most common localization (198–202). Moreover, pulmonary lymphomatoid granulomatosis, pulmonary Wegener's disease (203, 204), and systemic vasculitis (205, 206) have been described in XLP.

Most of these described diseases occur with EBV infection, and some authors have suggested that an impaired immune response to EBV can result in systemic vasculitis (206). However, EBV infections are not essential for the development of all XLP manifestations, including vasculitis (206).

## 6 Conclusions

In this review, we describe and discuss monogenic autoinflammatory disease, in which vasculitis is a prominent feature. Although the exact mechanisms are not always clear, they may be due to the direct cytotoxicity of endothelial cells due to IL-1ß, type I interferons, and probably immune complexes. The vasculopathy may involve either a small medium, or variable vessel and, rarely, a large vessel.

In a child presenting with vasculitis associated with increased inflammatory markers, particularly in cases of early onset of symptoms or positive family history, a monogenic autoinflammatory disease must be suspected, and a broad genetic test performed. In some cases, signs and symptoms, specific to each disease, may help direct the diagnosis and the genetic test. However, this is not always possible given the partial overlap of clinical manifestations among these conditions. Thanks to the new techniques, it is now possible to carry out the simultaneous analysis of a number of genes involved in SAIDS. This is particularly relevant given the importance of an early diagnosis. Vascular involvement may be as prominent in monogenic SLE as some PID. Clinicians should be aware of such vasculitic manifestations of autoinflammatory and autoimmune syndromes in order to establish a diagnosis and institute an appropriate treatment that, in some cases, may be lifesaving.

## Author contributions

SF, BLC, and FT: writing – original draft, writing – review and editing. RC, VL, GG, SV, RC, LL, SC, AS, CC, AI, MG, FB, GM, MG, and FC: writing – review and editing.

## Funding

The authors declare that no financial support was received for the research, authorship, and/or publication of this article.

## Conflict of interest

The authors declare that the research was conducted in the absence of any commercial or financial relationships that could be construed as a potential conflict of interest.
